# The brain slice method for studying drug distribution in the CNS

**DOI:** 10.1186/2045-8118-10-6

**Published:** 2013-01-21

**Authors:** Irena Loryan, Markus Fridén, Margareta Hammarlund-Udenaes

**Affiliations:** 1 Department of Pharmaceutical Biosciences, Translational PKPD Research Group, Uppsala University, Associate member of SciLife Lab, Box 591, Uppsala, SE-75124, Sweden; 2 AstraZeneca Research and Development, Respiratory and Inflammatory Innovative Medicines, Department of Drug Metabolism and Pharmacokinetics, Mölndal, Sweden

**Keywords:** Brain slice method, Unbound volume of distribution in the brain, Neuropharmacokinetics, Drug discovery, High-throughput screening

## Abstract

The high-throughput brain slice method is a precise and robust technique for estimating the overall uptake of drugs into brain tissue through determination of the unbound volume of distribution in the brain (V_u,brain_; ml·g brain^-1^). V_u,brain_ describes the relationship between the total drug concentration in the brain and the concentration of unbound drug in the brain interstitial fluid, regardless of blood–brain barrier function. The brain slice method is more physiologically based than the brain homogenate method with respect to the assessment of drug distribution in the brain because the cell-cell interactions, pH gradients and active transport systems are all conserved. The method provides information that is directly relevant to issues such as nonspecific binding to brain tissue, lysosomal trapping, and active uptake into the cells. For these reasons, the brain slice method is recommended for estimation of target-site pharmacokinetics in the early drug discovery process and fundamental pharmacological studies. This article provides a detailed protocol for the rat and mouse brain slice methods, with the aim of enabling simple, cost-effective profiling of compounds with diverse physicochemical properties. The procedure for assessing the viability of the brain slices after the 5 h incubation period is also described. The results are interpreted for a set of compounds covering a wide range of physicochemical properties and various pharmacological targets. Application of the method for evaluating the unbound intracellular-to-extracellular concentration ratio (K_p,uu,cell_) and the unbound brain-to-plasma concentration ratio (K_p,uu,brain_) is discussed.

## Background

It is generally accepted that the cerebral concentration of unbound drug is the main pharmacokinetic determinant of CNS activity for neurotherapeutics [1-3]. Preliminary assessment of the clinically relevant pharmacokinetic parameters required for approximation of the unbound-drug concentration in the brain interstitial fluid is thus pivotal in guiding early drug discovery research [4]. Because of the cost and complexity of the methodology, many of the available “gold standard” pharmacokinetic methods are not appropriate for use in the early stages of drug discovery. Consequently, there is an urgent need for adequate high-throughput in vitro systems and methods for CNS drug development programs.

The implementation of the high-throughput equilibrium dialysis-based assay for estimation of the fraction of unbound drug in the brain tissue (f_u,brain_), combined with measurement of whole brain concentrations in vivo*,* was groundbreaking for the field [5]. However, homogenization of the brain as used in this method changes the brain tissue binding properties, leading to implicit errors in the readouts [6].

In contrast, the brain slice method has a more physiological basis and has several benefits over the brain homogenate method. The brain slice preparation method was implemented by Henry McIlwain and is now extensively used in neurobiology, biophysics and quantitative pharmacology [7-9]. It has the advantage of offering a strongly regulated in vitro environment, while preserving much of the complex cellular integrity, including cellular barriers and intact circuitry, and as a result conserving functionality – resulting in an in vitro environment more comparable to the in vivo brain than seen in the homogenate method.

Several research groups have used the method to estimate the uptake of exogenous compounds into the brain [10-15]. Moreover, studies have investigated mechanistic pharmacokinetic/pharmacodynamic links using brain slice methodology [13,16].

Measurements obtained from in vivo microdialysis have also been compared to those from the in vitro brain slice and homogenate methods [17]. The reasonable correspondence (within a 3-fold range) between the cerebral microdialysis and brain slice method results in this study indicates that the brain slice method is the choice of preference [17].

The brain slice method has recently been further developed for high-throughput, making it more accessible for use by pharmaceutical companies [18]. It is now a precise, robust technique for estimating the overall uptake of drugs into brain tissue through determination of the unbound volume of distribution in the brain (V_u,brain_; ml·g brain^-1^). V_u,brain_ describes the relationship between the total drug concentration in the brain and the unbound-drug concentration in the brain interstitial fluid, regardless of blood–brain barrier function. The key assumption of the experiment is that, at equilibrium, the unbound-drug concentration in the brain slice interstitial fluid (ISF) or extracellular fluid (ECF) is equal to the drug concentration in the buffer in the beaker.

This article provides a detailed protocol for the rat and mouse brain slice methods, with the aim of encouraging simple, cost-effective profiling of compounds with diverse physicochemical properties and unifying procedures among laboratories in order to aid the achievement of comparable results.

## Methods and Design

### Animals

The protocols presented below are based on animal experiments approved by the Animal Ethics Committee of Uppsala, Sweden (C21/9 and C351/11). Drug-naïve male Sprague–Dawley 250–300 g rats and Naval Medical Research Institute (NMRI) 25–30 g mice were used (Taconic, Lille Skensved, Denmark). The fresh brain slices can be prepared from various strains of wild type and genetically modified mice and rats, depending on the purpose of the study and the respective laboratory traditions. The brain slices could as well be genetically manipulated using methods such as viral infection [19], biolistics [20], etc.

### Preparatory steps

#### Artificial extracellular fluid

To ensure maintenance of the brain slices in a healthy state, the artificial settings should mimic the in vivo cellular environment. The composition of artificial cerebrospinal fluid or extracellular fluid (aECF) is crucial. A large number of formulations for these artificial fluids can be found in the literature. In the experimental settings used in the studies underlying this paper, the HEPES-buffered aECF contained 129 mM NaCl, 3 mM KCl, 1.4 mM CaCl_2_, 1.2 mM MgSO_4_, 0.4 mM K_2_HPO_4_, 25 mM HEPES, 10 mM glucose and 0.4 mM ascorbic acid [18]. Ascorbic acid is used as a natural free-radical scavenger to protect the cell membranes from lipid peroxidation and the brain slices from swelling [21].

Prior to starting an experiment, a stock solution of aECF (1290 mM NaCl, 30 mM KCl, 12 mM MgSO_4_, 4 mM K_2_HPO_4_, 250 mM HEPES) is prepared and stored at room temperature. The 400 mM stock solution of ascorbic acid should be stored at +4°C.

On the day before the experiment, 1 L of Milli-Q water should be dispensed. On the day of experiment, this is used to prepare the working aECF solution according to the formulation (Table 1). The solution is then equilibrated with 100% oxygen for 15 minutes in an ice-water bath. The pH of the aECF should be 7.6 at 23°C at the beginning of the experiment and about 7.3 at 37°C directly after the 5 h incubation. See Table 2 for a summary of the critical steps in the brain slice experiment protocol.

**Table 1:** Composition of the working aECF solution

	**Weight (g)/volume (ml)**
Glucose	1.802 g
Milli-Q water*	600 ml
Stock aECF	100 ml
280 mM CaCl_2_	5 ml
400 mM Ascorbic acid	1 ml
pH (23°C)	**Adjust pH to 7.6** (up to 1.5 ml of 10 M NaOH)
Milli-Q water	Adjust volume to 1000 ml

* should be dispensed the day before the experiment.

**Table 2:** Critical steps in the brain slice experiment

**Experimental stages**	**Critical steps**
**Preparatory steps**	Control the pH, osmolarity and oxygenation of the aECF
	Take into account the pK_a_ values of the compounds when selecting the drugs to be investigated in one cassette
	Do not take longer than 1 minute to extract the brain
	Keep cold-chain during the brain slicing procedure
	Preserve the brain slices in ice-cold oxygenated aECF before starting the incubation
**Incubation**	Keep oxygenation, temperature and stirring constant during the incubation
**Preparation of the samples for bioanalysis**	Make sure all minor debris from the brain slices has sedimented before taking the aECF samples after the 5 h incubation

#### Preparation of cassettes

This protocol allows simultaneous investigation of a selection of up to ten compounds within the same experiment, allowing coverage of a wide range of physicochemical properties and various pharmacological targets in the same cassette.

When deciding on the compounds of each cassette, the pK_a_ values of the compounds should be taken into account. Because high concentrations of weak bases can increase the pH of the acidic intracellular compartments, the extent of lysosomal trapping of a weak base could be affected by the existence of another weak base. The interaction between two weak bases is mainly regulated by the concentrations of the free compounds in the cassette and their potency in increasing intralysosomal pH [22]. Consequently, it is recommended that the final aECF concentration of each studied compound in the cassette should be 100–200 nM and the total concentration of the studied compounds should not exceed 1 μM [18].

Each cassette of compounds is prepared individually, *ex tempore,* in scintillation vials (20 ml glass vials with screw lids; one vial per rat or mouse brain). Initially, the required volume of stock drug solution is added to an empty scintillation vial. To reduce possible toxic effects of the solvents (methanol, acetonitrile, etc.) on the brain slices, the solvents are evaporated under a gentle stream of nitrogen before diluting the sample with aECF. When using dimethyl sulfoxide (DMSO) to dissolve the compounds, it is strongly recommended that the final concentration of DMSO is kept as low as possible (no higher than 1%). Subsequently, 20 ml of ice-cold aECF, pre-equilibrated with 100% oxygen, is added to each scintillation vial and ultrasonicated for 10 minutes to facilitate the dissolution of the compounds. These ready-to-use solutions are maintained at 37°C until incubation.

#### Preparation of slices

The glassware and tools are set up for the dissection prior to preparing the brain slices. The vibrating blade microtome (e.g. Leica VT1200 (Leica Microsystems AB, Sweden)) is prepared for slicing and the chambers are chilled.

Drug-naïve rats/mice are anesthetized with inhalation anesthesia using 5% isoflurane. When deep anesthesia is reached, up to 10 ml of blood (rats) is collected intracardially. The animal is then decapitated and the skull quickly opened. The isolated brain is immediately placed into blank ice-cold aECF saturated with oxygen. In our experience, the brains should be sliced within 15–20 minutes of harvesting to retain their viability. Slices from three rat/mouse brains are generally equilibrated during the same day with one cassette of drugs.

The pre-chilled chamber of the vibratome is filled with ice-cold oxygenated aECF just before use and is then placed in the ice-filled tray of the vibratome.

It is advantageous to put one or two drops of cyanoacrylate glue on the cutting platform one minute before mounting the brain, to allow the glue to dry slightly.

**Figure 1:**
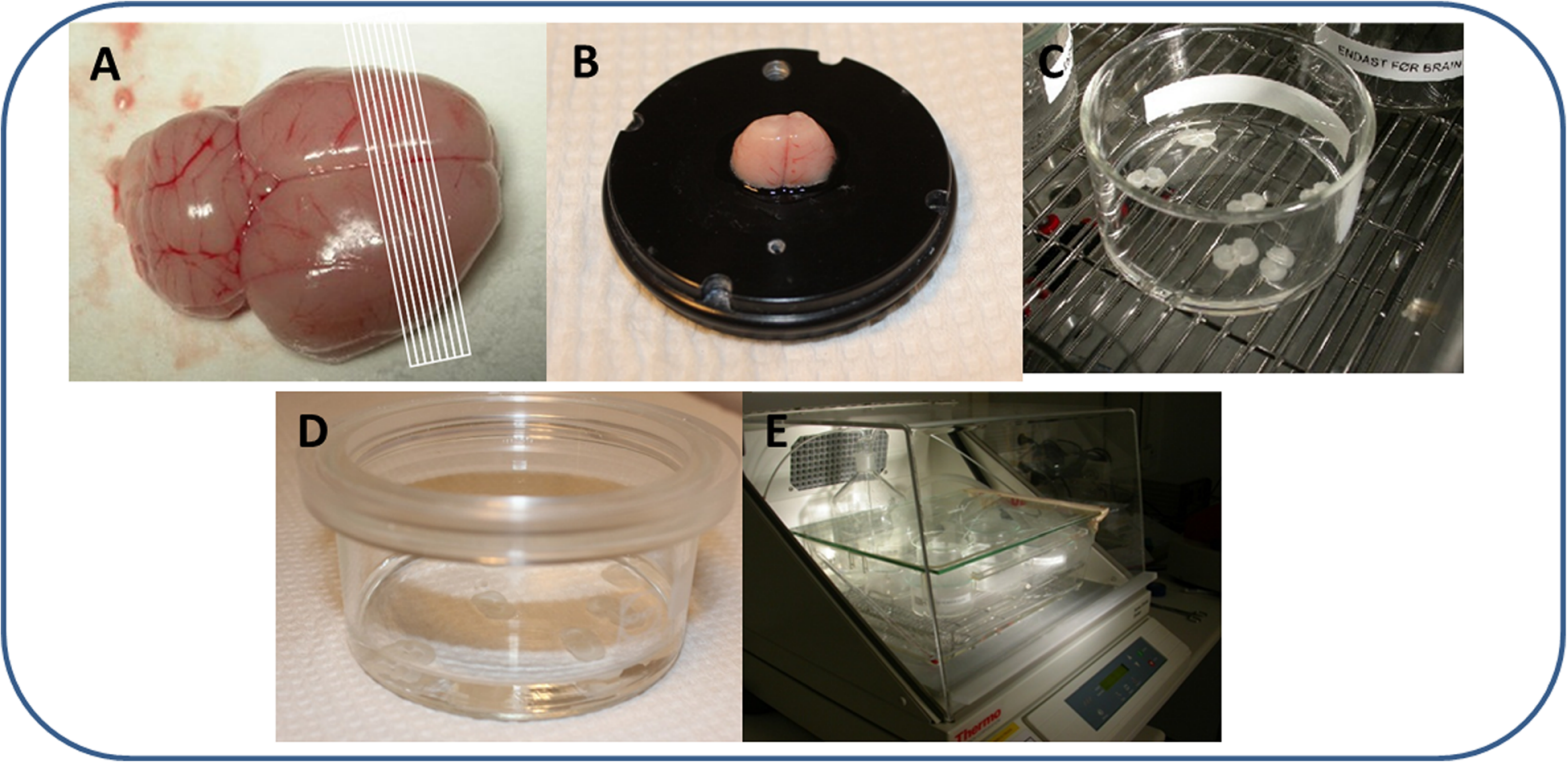
**The main steps in the preparation of brain slices. A**. Schematic representation of the cutting directions. **B**. Brain glued to the slicing platform in a coronal position. **C**. Brain slices transferred into the 80 mm diameter, flat-bottomed glass beaker. **D**. Beaker covered by custom-fabricated lid composed of a Teflon fluorinated ethylene-propylene film. **E**. Setup for the incubation-equilibration period.

Working quickly, the brain is placed on a chilled Petri dish covered with filter paper. Using a #22 surgical blade, a 3 mm piece is cut from the rostral area on a coronal plane, leaving a piece of about 10 mm for subsequent slicing. A caudal cut is then made (Figure 1A). The 10 mm piece of the brain is glued to the slicing platform in a coronal position (Figure 1B), and the platform is then positioned in the slicing chamber filled with the blank ice-cold aECF. The razor blade (Gillette, super-stainless) is then mounted and the clearance angle is fixed at 21°. We use a motorized blade holder sectioning speed of 0.8 mm/s with a 1 mm amplitude, in 0.05 mm steps.

After discarding the first one or two brain slices, 6 (rat brain) or 10 (mouse brain) consecutive 300 μm brain slices are cut on a coronal plane, starting approximately 1.7 mm anterior to the bregma (rostral striatum).

The 300 μm thickness provides good cell preservation without compromising the diffusion of oxygen into the core of the slice. The equilibration time during incubation is inversely related to the square of the brain slice thickness [16].

The slices are moved, using a micro spatula, to the brain slice storage beaker filled with oxygenated blank aECF which is kept in an ice bucket before the incubation. It is recommended that only brain slices with intact edges be used for the experiment, to reduce the amount of debris detaching from the brain tissue during incubation.

The slicing platform should be scrubbed to remove the brain and glue remnants, and the chamber refilled with fresh ice-cold oxygenated aECF, before proceeding with the next brain. The vibratome chambers should be cleaned, disinfected and dried at the end of each experimental day.

### Incubation

The incubation-equilibration process is started by gently transferring the 6 (rat) or 10 (mouse) brain slices from the storage beaker (using a micro double-ended spatula) into one 45 mm high, 80 mm diameter, flat-bottomed glass beaker (Duran Group, VWR, Sweden) containing 15 ml (rat) or 10 ml (mouse) of the aECF containing the selection of drugs to be investigated (Figure 1C). The beaker is then filled with humidified 100% oxygen over the aECF and covered with a custom-fabricated lid (Figure 1D) composed of a Teflon fluorinated ethylene-propylene (FEP) film (50 Å, 12.7 μm thick; DuPont, Katco Ltd, UK) as designed by Potter and DeMarse with minor modifications [23]. A “blank” beaker is also incubated in parallel to check stability of the compounds added into the buffer.

The transparent Teflon FEP film is used in preference to a glass lid because it is selectively permeable to gases (e.g. oxygen) while remaining relatively impermeable to water vapor. This significantly decreases evaporation (allowing improved control of osmolarity and pH), thus permitting the use of a non-humidified incubator.

Finally, the beaker is placed inside the small plastic box in the incubated shaker (e.g. MaxQ4450 Thermo Fisher Scientific, NinoLab, Sweden) for 5 h at 37°C (Figure 1E). Control of the temperature by an external thermometer is recommended. A rotation speed of 45 rpm and oxygen flow of about 75–80 ml per minute through a glass frit apparatus appear sufficient.

The pH of the aECF should be measured at 37°C immediately after the 5 h incubation. A reduction of more than 0.15 pH units over the 5 hours indicates more than acceptable acidification of the buffer.

### Preparation of the samples for bioanalysis

It is necessary to take several samples for bioanalysis during the experiment:

I. aECF samples

a. for thermostability testing of the compounds studied (sampled before and after incubation in aECF without the brain slices)

b. for measurement of C_buffer_ – the final concentrations of the unbound compounds in aECF (sampled at the end of the 5 h incubation with the brain slices)

II. Brain slices samples

a. for measurement of A_brain_ – the amount of drug in the brain slices (sampled at the end of the 5 h incubation)

Procedural details for preparation of the aECF and brain slice samples are given below.

I. aECF samples

a. Assessment of the thermostability of the compounds provides valuable information and is recommended for each drug selection tested. A 200 μl sample of aECF is taken directly from the beaker at the beginning of the study and at the end of 5 h incubation without brain slices for analysis of initial and final concentrations of the drugs in aECF. The aECF sample is transferred into an Eppendorf tube containing 200 μl blank brain homogenate that has been prepared beforehand with 4 volumes of blank aECF. The brain homogenate is included to prevent nonspecific binding of drugs to the plastic as well as to match the matrix of the slice homogenates, as required for the following LC-MS-MS analysis.

b. Since the unbound drug concentration in the brain slice interstitial fluid at equilibrium is taken to be equal to the drug concentration in the aECF in the beaker, the presence of any minor debris from the brain tissue should be avoided in the sampled aECF after the 5 h incubation. To achieve this, the beaker needs to sit still for 5 minutes after finishing the incubation before sampling the aECF. To sample, 200 μl of the aECF is aspirated from just below the surface (without wetting the tip before sampling) and dispensed into an Eppendorf tube containing the 200 μl of blank brain homogenate, as described in Ia. It is recommended that duplicate samples of the aECF be taken at this stage. Filtration of aECF, which is recommended by several authors, is not suitable for drug distribution studies because of the possible loss of the compounds in the filter.

II. Brain slice samples

a. After sampling the aECF, the brain slices in the aECF are individually removed, dried on filter paper (about 30 s), weighed (~33 mg per rat brain slice and ~13 mg per mouse brain slice) and homogenized separately in 9 volumes (w/v) of aECF with an ultrasonic processor (e.g. VCX-130; Sonics, Chemical Instruments AB, Sweden).

The samples are then stored at −20°C pending bioanalysis (e.g. LC-MS-MS).

### Assessment of viability of brain slices

The brain slices must remain viable during the experiment. There are several methods of assessing viability based on biochemical and/or electrophysiological parameters. In our laboratory, the viability of the brain slices is assessed by measuring the relative activity of released lactate dehydrogenase (LDH) using a cytotoxicity detection kit [24] according to the manufacturer's instructions (Roche Diagnostics GmbH, Germany), with some modifications.

A brief description of the preparation of the main controls and samples required for analysis is given in Tables 3 and 4.

**Table 3:** Brief description of assessment of viability of brain slices based on the activity of released lactate dehydrogenase

1.	Preparation of **Background** absorbance control. Take 200 μl of aECF buffer from the scintillation vial containing the drug cassette before incubation and mix with 200 μl of blank aECF (store at +4°C pending analysis)
2.	Preparation of **Low control.** Take 200 μl of aECF buffer from the beaker containing the drug cassette five minutes after transferring the freshly prepared brain slices into the beaker and mix with 200 μl of blank aECF buffer (store at +4°C pending analysis)
3.	Preparation of **Samples.** Take 200 μl of aECF buffer from the beaker containing the drug cassette and brain slices after 5 hours of incubation and mix with 200 μl of blank aECF buffer (store at +4°C pending analysis)
4.	Preparation of **High control.** Place one (rat) or 3 (mouse) weighed brain slices in an Eppendorf tube after 5 hours of incubation with the drug cassette, and add 9 volumes (w/v) of 2% Triton X-100 solution in aECF. Put Eppendorf tubes in ultrasound bath for 1 hour at +4°C. Then incubate the tubes for 30 minutes in a water bath at 37°C. Centrifuge at 10000 rpm for 5 minutes at +4°C Take supernatant and store at +4°C pending analysis
5.	Preparation of the samples for analysis, see Table 4. Perform all tests **in triplicate** Protect the plate from light after addition of freshly prepared reaction mixture
6.	Incubate the plate for up to 25 minutes at room temperature
7.	Measure the absorbance of the samples at 492 nm (use 690 nm as a reference wavelength)

**Table 4:** Preparation of samples for assessment of viability of brain slices based on the activity of released lactate dehydrogenase

	**aECF buffer**	**Low control**	**Sample**	**High control**	Reaction mixture*
Background	100 μl				100 μl
Low control		100 μl			100 μl
Sample			100 μl		100 μl
High control				100 μl	100 μl

* The reaction mixture should be prepared *ex tempore* according to the manufacturer's instructions.

To calculate the viability of the brain slices (as a percentage) after the 5 h incubation-equilibration period, the following steps are taken for each experimental setup:

•Background absorbance control - provides information about the background absorbance of the assay medium (aECF). For this purpose, 200 μl sample of aECF is taken at the beginning of the experiment from the scintillation vial and mixed with 200 μl blank aECF. The obtained absorbance value is then subtracted from all other values.

•Low control - provides information about the activity of LDH released from the brain slices as a result of damage to the membranes caused by slicing the brain. A 200 μl aECF sample is taken 5 minutes after transferring the brain slices into the beaker for incubation and is then mixed with 200 μl blank aECF.

•High control - provides information about the maximum possible activity of releasable LDH in the brain slice. To achieve this, one rat or three mouse brain slices are used after the 5 h incubation for each experimental setup. After drying the brain slice(s) on filter paper and individually weighing them, 9 volumes (w/v) of 2% Triton X-100 solution in aECF is added. To facilitate the release of LDH from the brain slice(s), the Eppendorf tube is placed in an ultrasound bath for one hour at +4°C followed by 30 minutes of incubation in a water bath at 37°C. The supernatant obtained after centrifugation of the tube for 5 minutes at 10,000 rpm and +4°C is stored at +4°C pending analysis (no longer than 5 days).

For preparation of the experimental samples (experimental value), 200 μl of aECF sample is taken after the 5 h incubation-equilibration period and mixed with 200 μl blank aECF. For evaluation of the effects of changes in viability of the brain slices during the incubation, aECF samples can be taken at different time points (after 1, 2, 3, etc. hours).

Once the absorbance of the control and experimental samples has been obtained (Table 3) the relevant viability (%) of the brain slices can be calculated according to Equations 1 and 2:

(1) $$\text{Cytotoxicity}\left(\%\right)=\frac{\text{exp}\text{erimental}\;\text{value-low}\;\text{control}}{\text{high}\;\text{control-low}\;\text{control}}\times 100$$

(2) $$\text{Viability}\left(\%\right)=100\%-\text{Cytotoxicity}\left(\%\right)$$

In practice, it is recommended that 85-90% viability be aimed for; however, a viability of around 60% provides similar results according to our experience (data not shown). Viability values lower than 50% after the 5 h incubation period are associated with dramatic changes in the estimation of V_u,brain_, especially for weak bases and results from the experiments should be discarded.

### Estimation of V_u,brain_

Bioanalytically determined drug concentrations in the brain slices and the 5 h aECF samples are used to estimate V_u,brain_. It is critical to remember to scale back the obtained concentrations (or areas under the concentration-time peaks) to the undiluted buffer and brain slice concentrations by multiplying by the dilution factors as appropriate. The concentration in each brain slice sample is multiplied by 10 to account for the dilution during preparation of the homogenate. The concentration in aECF is multiplied by 2 to account for the dilution during 1:1 mixing of the aECF sample with blank brain homogenate (in 4 volumes (w/v) of aECF). The dilutions associated with protein precipitation are not accounted for because they are the same for all samples.

V_u,brain_ (ml · g brain^-1^), as defined in Equation 3, is equivalent to the ratio of the amount of drug in the brain or brain slice (A_brain_, nanomoles · gram brain^-1^) to the measured final aECF concentration (C_buffer_, micromoles per liter) or unbound brain interstitial fluid concentration (C_u,brainISF_) measured using cerebral microdialysis technique:

(3) $$V_{u,\text{brain}}=\frac{A_{\text{brain}}}{C_{u,\text{brainISF}}}=\frac{A_{\text{brain}}}{C_{\text{buffer}}}$$

Because of incomplete absorption of aECF by the filter paper, the brain slices have a surrounding layer of aECF, and it is important to measure the volume of this layer (V_i_, milliliters per gram of slice) and compensate for this aECF buffer layer, i.e. (1-V_i_) in Equation 4. V_i_ should be measured in a separate experiment using [^14^C] inulin as described in Fridén et al. [18]. V_i_ was reported to be 0.094 ml ∙g slice^-1^[18]. In view of this, Equation 3 can be reorganized to obtain V_u,brain_ corrected for the remaining aECF volume on the brain slice:

(4) $$V_{u,\text{brain}}=\frac{A_{\text{brain}}-V_{i}\cdot C_{\text{buffer}}}{C_{\text{buffer}}\cdot \left(1-V_{i}\right)}$$

### High-throughput screening capacity

Once the brain slice methodology is established in the laboratory (Table 5), it can be used in a high-throughput manner. One trained individual can perform up to four experiments per day (using rats or mice). The method allows up to 10 compounds to be tested simultaneously (consultation with an analytical chemist is required). A series of three experiments is enough to obtain consistent results for one cassette.

**Table 5:** Checklist before starting the experiments

**aECF**	Stock aECF
	280 mM CaCl2
	400 mM ascorbic acid
	Dispensed the day before 1 L MQ water
	Oxygen supply
	pH meter
**Apparatus**	Orbital shaking incubator
	Vibratome
	Nitrogen evaporator
	Ultrasonic bath
	Ultrasonic processor
	Centrifuge
	Water bath
	ELISA reader
**Glass- and lab-ware**	Petri dishes
	45 mm high, 80 mm diameter, flat-bottomed glass beakers
	Custom-fabricated lids of Teflon FEP film for beakers
	Scintillation vials
	Surgical instruments for dissection of brain
	Ice buckets
	Pre-labeled Eppendorf tubes
	Nunc 96-well plates
	Blank brain homogenate (in 4 volumes (w/v) of aECF)
**Miscellaneous**	Cyanoacrylate glue
	Cytotoxicity detection kit

## Discussion

**Figure 2:**
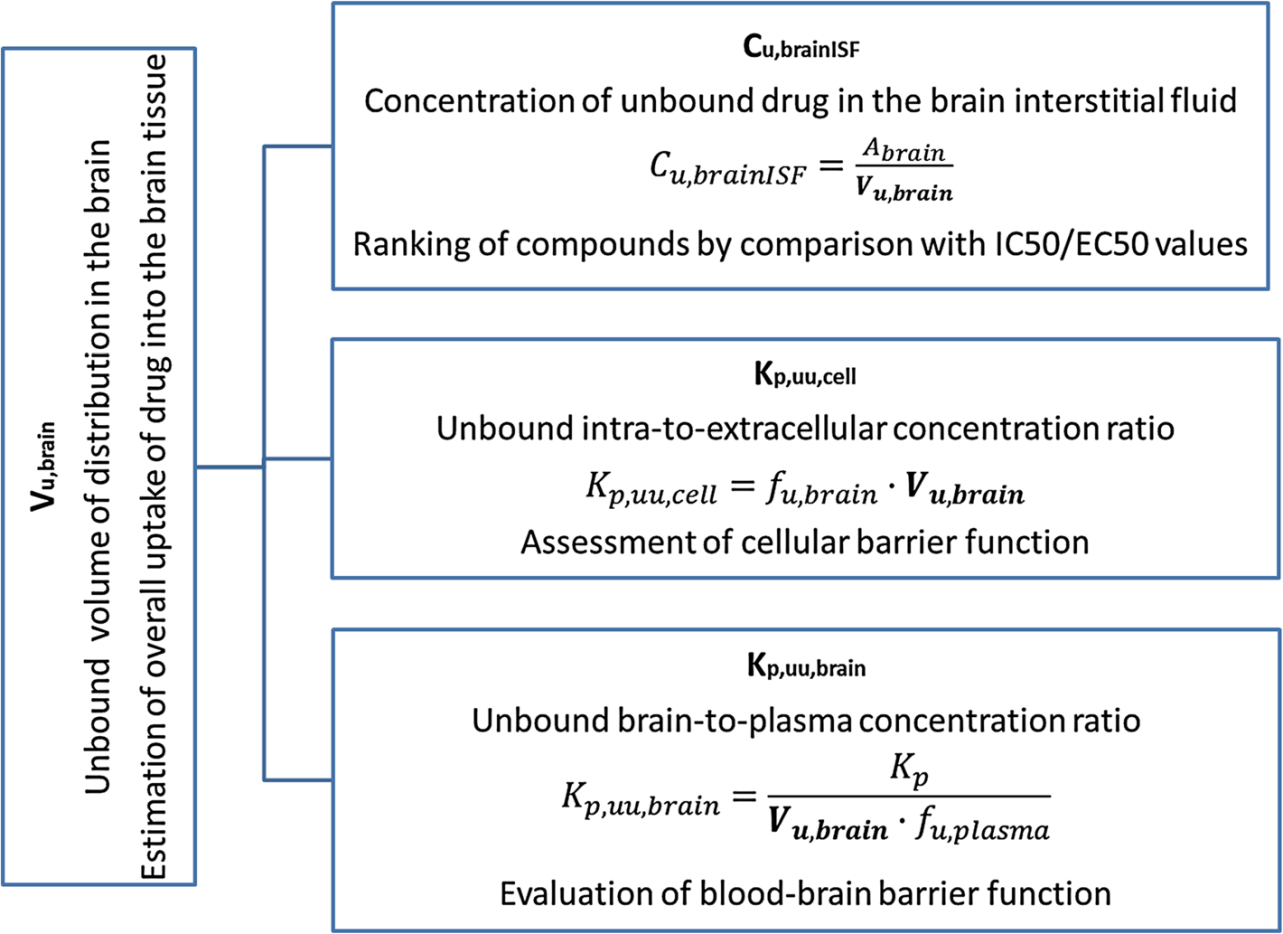
**A number of applications of V**_**u,brain **_**for integrative pharmacology.**

The high-throughput rat or mouse fresh brain slice method is a powerful tool for estimating the intracerebral distribution of diverse compounds in an in vitro setup with preserved cellular barrier functionality. The method allows the estimation not only of nonspecific binding of compounds to the brain tissue but also of the cellular accumulation of compounds through uptake transporters, trapping in acidic intracellular compartments (i.e. lysosomes), and active efflux from the cellular membrane [6]. Consequently, the determination of more physiological V_u,brain_ values using fresh brain slices instead of brain homogenates permits more precise assessment of C_u,brainISF_ (Figure 2) with reduced risk of misrepresentation during subsequent evaluation of exposure-target engagement relationships.

The estimated V_u,brain_ value can be put into context by comparison with the physiological volume of brain fluids, e.g. V_u,brain_ values higher than 0.8 ml · g brain^-1^ (the volume of total brain fluids is 0.8 ml · g brain^-1^) are interpreted as intracellular distribution of the drug in question [4,18]. Values higher than this indicate that proportionally more of the drug is intracellularly distributed, e.g. into lysosomes, and/or bound to parenchymal components. A value lower than 0.8 ml · g brain^-1^ shows restricted distribution. The lowest volume possible in vivo is the brain interstitial fluid volume of 0.2 ml · g brain^-1^; however, in the slices, this is somewhat higher because of damage to the surface layer of cells.

Table 6 shows V_u,brain_ values of nine drugs covering a wide range of physicochemical properties and pharmacological targets. V_u,brain_ has extensive variability with a range from 3.75 to 2650 ml · g brain^-1^.

**Table 6:** **Unbound volume of distribution in the brain (V**_**u,brain**_**) determined using Sprague–Dawley (SD) rat and Naval Medical Research Institute (NMRI) mouse brain slices**

	**Ion class**	**V**_**u,brain **_***ml·g brain***^***-1***^	
		**SD rat**	**NMRI mouse**
Verapamil	Base	46.6 (1.8)	47.3 (4.4)
Docetaxel	Base	777 (217)	796 (177)
Oxycodone	Base	4.20 (0.13)*	3.75 (0.22)
Digoxin	Base	33.1 (6.0)	44.9 (5.7)
Gabapentin	Zwitterion	4.49 (0.29)	4.22 (0.93)
Indomethacin	Acid	14.1 (1.8)	12.0 (1.8)
Paroxetine	Base	714 (72)	596 (97)
Thioridazine	Base	2650 (232)	1930 (170)
Diazepam	Neutral	17.8 (1.1)	17.1 (2.3)

* Data from Fridén et al., 2011 [6].

Data are reported as means (standard deviations).

An additional very important aspect of drug discovery is the ability to extrapolate the results to other species. Recently, it has been shown that unbound fraction of drug in brain homogenate value obtained from the *Wistar Han* rat brain homogenate can be used as a representative value for any preclinical species and also humans [25]. The results in Table 6 indicate an absence of any significant dissimilarity in V_u,brain_ values between Sprague–Dawley rats and NMRI mice. However, a more systematic investigation is desirable before the possibility of interchangeable use of V_u,brain_ measurements can be supported for translational studies.

It has been proposed by Fridén et al. [17] that in vitro determination of V_u,brain_ in combination with in vivo determination of the total brain-to-plasma concentration ratio (K_p,brain_) and in vitro determination of the fraction of unbound drug in plasma (f_u,plasma_) would allow rapid evaluation of unbound brain-to-plasma concentration ratio (K_p,uu,brain_) (Figure 2). Moreover, combining V_u,brain_ with f_u,brain_ would allow estimation of the unbound intracellular-to-extracellular concentration ratio (K_p,uu,cell_). It is essential to emphasize that, in terms of predicting C_u,brainISF_ or K_p,uu,brain_, rank ordering of compounds with respect to V_u,brain_ is futile, since there is no causal relationship [4].

Complex evaluation of the abovementioned neuropharmacokinetic parameters gives insight to drug distribution in the brain. For instance, analogue of γ-aminobutyric acid gabapentin has K_p,brain_ equal to 0.64 [26]. However, after correction of K_p,brain_ for brain tissue uptake (using V_u,brain_ derived from the brain slice method) and plasma protein binding (using f_u,plasma_) the BBB net flux was estimated as a 0.14 meaning that only 14% of unbound drug in plasma is passing the BBB. Moreover, after passing BBB gabapentin (substrate to the large neutral amino acid transporter) tends to accumulate in the cells as it could be judged from K_p,uu,cell_ equal to 4.55 [6].

The brain slice method can also be used to identify suitable positron emission tomography (PET) tracers, which should have a low degree of nonspecific binding (i.e. a low V_u,brain_ value) to obtain higher specificity for their targets.

In summary, the brain slice method, used for assessment of the volume of distribution of unbound drug in the brain, is a useful tool for both drug discovery and fundamental pharmacology research.

## Abbreviations

aECF: Artificial extracellular fluid; Abrain: Amount of drug in brain tissue; BBB: Blood–brain barrier; Cbuffer: Final drug concentration in aECF; Cu,brainISF: Concentration of unbound drug in the brain interstitial fluid; CNS: Central nervous system; DMSO: Dimethyl sulfoxide; ECF: Extracellular fluid; HTS: High-throughput screening; fu,brain: Unbound fraction of drug in brain homogenate; fu,plasma: Unbound fraction of drug in plasma; Kp,brain: Total brain-to-plasma concentration ratio; Kp,uu,brain: Unbound brain-to-plasma concentration ratio; Kp,uu,cell: Unbound intracellular-to-extracellular concentration ratio; LDH: Lactate dehydrogenase; Vu,brain: Unbound volume of distribution in brain

## Competing interests

Irena Loryan is funded by a post-doc stipend from Johnson&Johnson. Markus Fridén is an employee of AstraZeneca R&D. Margareta Hammarlund-Udenaes: no conflicts of interest or other issues.

## Authors’ contributions

I L: Writing of manuscript, development of slice method and viability test. M F: Feedback on manuscript, proofreading. Development of slice method for high throughput studies, development of pH partitioning method etc. M H-U: Feedback on manuscript, proofreading. All authors have read and approved the final version of the manuscript.

## Authors’ information

You may choose to use this section to include any relevant information about the author(s) that may aid the reader's interpretation of the article, and understand the standpoint of the author(s). This may include details about the authors' qualifications, current positions they hold at institutions or societies, or any other relevant background information. Please refer to authors using their initials. Note this section should not be used to describe any competing interests.
